# Amantadine Alleviates Postoperative Cognitive Dysfunction Possibly by Preserving Neurotrophic Factor Expression and Dendritic Arborization in the Hippocampus of Old Rodents

**DOI:** 10.3389/fnagi.2020.605330

**Published:** 2020-11-26

**Authors:** Jing Zhong, Jun Li, Cheng Ni, Zhiyi Zuo

**Affiliations:** ^1^Department of Anesthesiology, University of Virginia, Charlottesville, VA, United States; ^2^Department of Anesthesiology, Zhongshan Hospital Fudan University, Shanghai, China; ^3^Department of Anesthesia, National Cancer Center/National Clinical Research Center for Cancer/Cancer Hospital, Chinese Academy of Medical Sciences and Peking Union Medical College, Beijing, China

**Keywords:** amantadine, dendritic arborization, neurotrophic factors, old rodents, postoperative cognitive dysfunction

## Abstract

**Objectives:**

Amantadine has been shown to attenuate postoperative learning and memory dysfunction in young adult rats. However, postoperative cognitive dysfunction often occurs in elderly patients. We aimed to determine whether amantadine attenuated postoperative learning and memory dysfunction and whether these effects were associated with improved dendritic arborization in old rodents.

**Methods:**

Eighteen-month old male C57BL/6J mice or Fischer 344 rats were subjected to right carotid artery exposure (surgery) under isoflurane anesthesia. This age represents an early old stage in rodents. Carotid artery exposure was used to simulate commonly performed carotid endarterectomy in elderly patients. Amantadine was injected intraperitoneally at 25 μg/g once a day for 3 days with the first dose at 15 min before surgery. The animals were tested by Barnes maze and fear conditioning starting one week after the surgery. Hippocampus was harvested for Western blotting and Golgi staining.

**Results:**

Surgery and anesthesia impaired the learning and memory in old mice and rats. Surgery reduced the expression of brain-derived neurotrophic factor (BDNF) and glial cell line-derived neurotrophic factor (GDNF), dendritic arborization and spine density in the hippocampus of old rats. These effects were attenuated by amantadine. The effects of amantadine were blocked by intracerebroventricular injection of anti-BDNF antibody or anti-GDNF antibody.

**Conclusion:**

Surgery and anesthesia impaired learning, memory and dendritic arborization in old rodents that are age relevant to postoperative cognitive dysfunction. These effects may be attenuated by amantadine via preserving the expression of neurotrophic factors.

## Introduction

Postoperative cognitive dysfunction (POCD) affects millions of patients annually and is associated with increased hospital stay length and 1-year mortality (Newman et al., 2001; Monk et al., 2008). Age is a risk factor for POCD (Newman et al., 2001; Monk et al., 2008). Currently, effective and practical interventions for POCD have not been established and are urgently needed to improve the outcome of patients after surgery.

Amantadine is a low-affinity non-competitive N-methyl-d-aspartate (NMDA) receptor antagonist and was initially marked as an antiviral agent (Kornhuber et al., 1994). It is now proposed to treat many other diseases or conditions, such as Parkinson disease (Kulisevsky et al., 2018), dyskinesia (Hauser et al., 2018) and traumatic brain injury (Hammond et al., 2017). Amantadine may attenuate the symptoms of these diseases and improve cognition of patients with these diseases. Amantadine may also improve wakefulness and cognition of patients with stroke when it is used during the acute phase (Gagnon et al., 2020; Leclerc et al., 2020). However, the use of amantadine at more than 6 months after brain trauma did not improve the cognition of these patients (Hammond et al., 2018), possibly because it was too late after the injury for any beneficial effect to occur. It has been suggested that one possible mechanism for the beneficial effects of amantadine is to enhance the production of glial cell line-derived neurotrophic factor (GDNF) (Rocha et al., 2012; Zhang et al., 2014). GDNF is a trophic factor that has neuroprotective properties on dopamine neurons in multiple animal species (Grondin et al., 2018). GDNF can inhibit microglial activation and neuroinflammation (Rocha et al., 2012; Zhang et al., 2014). Neuroinflammation is considered as an underlying pathological process for POCD (Cao et al., 2012; Zhang et al., 2014; Zheng et al., 2017). Our study has shown that amantadine attenuates POCD in young adult rats possibly via maintaining GDNF levels (Rocha et al., 2012; Zhang et al., 2014) and sepsis-induced cognitive dysfunction via inhibiting neuroinflammation in young mice (Xing et al., 2018). However, the effects of amantadine on old animals after surgery have not been reported.

Brain-derived neurotrophic factor (BDNF) is well known for its potential to promote brain plasticity (Toh et al., 2018). BDNF might be a mediator for methylene blue-associated neuroprotection (Bhurtel et al., 2018). The combination of cognitive and physical exercise has the potential to generate synergistic benefits in cognitive function by significantly increasing BDNF levels in the blood (Miyamoto et al., 2018). Our studies have shown that carotid artery exposure, a surgical procedure, reduced BDNF (Fan et al., 2016; Gui et al., 2017). However, the role of BDNF in amantadine-induced protection is not known.

Neuroplasticity is the underlying processes for learning and memory (Dayan and Cohen, 2011). Structurally, these processes include changes of dendritic arborization (Gillani et al., 2010; Knutson et al., 2016). Isoflurane, sevoflurane and desflurane were found to significantly increase dendritic spine density on dendritic shafts of layer 5 pyramidal neurons in the cerebral cortex of neonatal rats (Briner et al., 2010). However, isoflurane did not change synaptic density in the hippocampus at 29 days after isoflurane exposure in old rats (Lin et al., 2012). Hepatectomy in old rats induces differential loss of neuronal dendritic spines in the hippocampus (Le et al., 2014). Our recent study has shown that laparotomy decreases dendritic arborization and spine density in young adult mice (Luo et al., 2020). These studies suggest that surgery impairs dendritic arborization. However, it is not clear how this impairment occurs.

On the basis of the above information, we hypothesize that amantadine attenuates POCD via maintaining GDNF and BDNF to preserve dendritic arborization and spine density in the hippocampus. To test these hypotheses, old rats or mice were subjected to right carotid artery exposure, a surgical procedure that is part of commonly performed carotid endarterectomy in elderly patients. Their learning and memory were tested. The expression of GDNF and BDNF and dendritic arborization in the hippocampus of old rats were examined.

## Materials and Methods

The animal protocol was approved by the institutional Animal Care and use Committee of the University of Virginia (Charlottesville, VA, United States). All animal experiments were carried out in accordance with the National Institutes of Health Guide for the Care and use of Laboratory Animals (NIH publications number 80–23) revised in 2011.

### Animal Groups

In the first experiment, 18-month old male C57BL/6J mice weighing 33 – 40 g from the National Institute of Aging (Bethesda, MD, United States) were randomly assigned to: (1) control group (not being exposed to surgery or any drugs), (2) surgery group (right carotid artery exposure), and (3) surgery plus amantadine group. Each group had 15 mice. One week later, these mice were evaluated by Barnes maze and then fear conditioning tests.

In the second experiment, 18-month old male Fischer 344 rats weighing 362–483 g from the National Institute of Aging were randomly assigned to the three groups described above in the first experiment. Their hippocampus was harvested 3 days after surgery for Western blotting.

In the third experiment, 18-month old male Fischer 344 rats were randomly assigned to: (1) control group, (2) surgery (right carotid artery exposure) plus amantadine plus anti-GDNF antibody group, (3) surgery plus amantadine plus anti-BDNF antibody group, and (4) surgery plus amantadine plus heat-denatured anti-GDNF antibody group. These rats were evaluated by Barnes maze and then fear conditioning tests one week later.

In the fourth experiment, 18-month old male Fischer 344 rats were randomly assigned to: (1) control group, (2) surgery (right carotid artery exposure), (3) surgery plus amantadine plus anti-GDNF antibody group, (4) surgery plus amantadine plus anti-BDNF antibody group, and (5) surgery plus amantadine plus heat-denatured anti-GDNF antibody group. Their brains were harvested for Golgi staining 3 weeks after the surgery.

### Anesthesia and Surgery

The surgery was a right carotid artery exposure. As we described before (Zheng et al., 2017), mice were anesthetized by 1.8% isoflurane delivered by an agent-specific vaporizer and carried by gases contained 30% oxygen. During the procedure, the mouse was kept at spontaneous respiration. A 1.5 cm midline neck incision was made after the mouse was exposed to isoflurane for at least 30 min. The soft tissues over the trachea were retracted gently. One-centimeter long right common carotid artery was carefully dissected free from other tissues. Particular care was taken to avoid damage to the vagus nerve. The wound was then irrigated with normal saline and closed with surgical suture. The surgical procedure was performed under sterile conditions and lasted around 15 min. After the surgery, all animals received a subcutaneous injection of 3 mg/kg bupivacaine.

All rats received isoflurane to induce anesthesia. After loss of righting reflex was achieved, 0.15 mg/kg buprenorphine hydrochloride (Recktt Benckiser Healthcare Ltd., Kingston-upon-Thames, United Kingdom) was injected subcutaneously. The rat was immediately intubated with a 14-gage catheter and mechanically ventilated with 100% oxygen by a CIV-101 ventilator (Columbus Instruments, Columbus, OH, United States) to maintain normal end-tidal carbon dioxide concentrations as monitored by a Datex infrared analyzer (Capnomac, Helsinki, Finland). Anesthesia was maintained by 1.8% isoflurane. The front of neck was meticulously shaved and disinfected with povidone iodine. Ten minutes after the induction of anesthesia, a 2.2-cm midline neck incision was made and the soft tissues over the trachea were retracted gently. The rest of the procedures were the same as those of mice. After the surgery, isoflurane concentration was reduced to 1.2%. After isoflurane anesthesia was stopped, the rats were allowed to breathe spontaneously and then extubated at the recovery of righting reflex.

The total duration of anesthesia lasted for 2 h in mice and rats, a clinically relevant duration of anesthesia. No response to toe pinching that was performed every 15 min was observed during the whole anesthesia period. During anesthesia, rectal temperature was monitored and maintained at 37°C with the aid of servo-controlled warming blanket (TCAT-2LV, Physitemp instruments Inc., Clifton, NJ, United States).

### Amantadine Application

Amantadine (A1260, Sigma-Aldrich, St. Louis, MO, United States) was dissolved in normal saline and injected intraperitoneally at 25 μg/g/day daily for three days with the first dose at 15 min before surgery. The amantadine dose was chosen based on previous studies (Kim et al., 2012; Zhang et al., 2014).

### Intracerebroventricular Injection of Antibodies

Rats received intracerebroventricular injection of 10 μl (200 μg/ml) rabbit polyclonal anti-GDNF antibody (catalog number: sc-328; Santa Cruz Biotechnology, Santa Cruz, CA, United States) or anti-BDNF antibody based on a previous study (Rocha et al., 2012; Zhang et al., 2014). Others received the injection of 10 μl heat-denatured (5 min at 100°C) anti-GDNF antibody as we did before [3]. Each rat received single injection of antibodies to the right lateral ventricle immediately at the end of the surgery. The intracerebroventricular injection was performed as described before with the aid of a stereotactic apparatus (SAS-5100, ASI Instruments, Inc., Warren, MI, United States) using the following coordinates: 0.4 mm posterior to bregma, 1.5 mm lateral from midline and 4.5 mm ventral from the surface of the skull. After the injection, the needle was kept in place for 1 min to prevent backflow of the injected solution.

### Barnes Maze

The animals were subjected to Barnes maze as we previously described to test their spatial learning and memory (Zheng et al., 2017). Animals were first placed in the middle of a circular platform with 20 equally spaced holes (SD Instruments, San Diego, CA, United States; the circular platforms for rats and mice are different in size). One of these holes was connected to a dark chamber called target box. Aversive noise (85 dB) and bright light (200 W) shed on the platform was used to encourage animals to find the target box. They had a spatial acquisition phase that lasted for 4 days with 3 min per trial, 4 trials per day and 15 min between each trial. Animals then went through the reference memory phase to test the short-term retention on day 5 and long-term retention on day 12. No test or handling was performed from day 5 to day 12. The latency to find the target box during each trial was recorded with the assistance of ANY-Maze video tracking system (SD Instruments).

### Fear Conditioning

One day after Barnes maze test, animals were subjected to fear conditioning test as we previously described (Zheng et al., 2017). Each animal was placed into a test chamber wiped with 70% alcohol and exposed to three tone-foot shock pairings (tone: 2000 Hz, 85 dB, 30 s; foot shock: 1 mA, 2 s) with an intertrial interval 1 min in a relatively dark room. The animal was removed from this test chamber 30 s after the conditioning stimuli. The animal was placed back to the same chamber without the tone and shock 24 h later for 8 min. The animal was placed 2 h later into another test chamber that had different context and smell from the first test chamber in a relatively light room. This second chamber was wiped with 1% acetic acid. Freezing was recorded for 3 min without the tone stimulus. The tone was then turned on for three cycles, each cycle for 30 s followed by 1-min inter-cycle interval (4.5 min in total). Animal behavior in these two chambers was video recorded. The freezing behavior in the 8 min in the first chamber (context-related) and 4.5 min in the second chamber (tone-related) was scored by an observer who was blind to the group assignment.

### Brain Tissue Harvest

Rats were deeply anesthetized with 5% isoflurane for 2 min and perfused transcardially with normal saline. Their hippocampus was dissected out immediately for Western blotting and their whole brain for Golgi staining. The perfusion was necessary to eliminate the contamination of blood cells in the brain tissues. The very short duration (in minutes) of perfusion and anesthesia processes shall not affect the expression of growth factors and neural structures.

### Western Blotting

Hippocampus was homogenized in a buffer (pH 7.9) containing 10 mM HEPES, 1.5 mM MgCl_2_, 10 mM KCl, 0.5 mM dithiothreitol, 0.05% NP40 and protease inhibitor cocktail (10 mg/ml aproteinin, 5 mg/ml peptastin, 5 mg/ml leupeptin, and 1 mM phenylmethanesulfonylfluoride) and incubated on ice for 10 min. They were then centrifuged at 13,000 rpm for 20 min at 4°C. The supernatant was kept for Western blotting.

Fifty microgram of proteins per lane were separated on a polyacrylamide gel and then blotted onto a polyvinylidene difluoride membrane. The membranes were blocked with protein-free T20 blocking buffer (catalog number: 37573, Thermo Scientific, Logan, UT, United States) and incubated with the following primary antibodies overnight at 4°C: rabbit polyclonal anti-GDNF antibody (1:200 dilution, catalog number: sc-328; Santa Cruz Biotechnology), rabbit polyclonal anti-BDNF antibody (1:200 dilution, catalog number: sc-546; Santa Cruz Biotechnology) or mouse monoclonal anti-β-actin (1:5000 dilution, catalog number: ab6276; Abcam). Protein bands were captured by Genesnap version 7.08 and quantified by Genetools version 4.01.

### Golgi Staining

The study was performed according to the manufacturer’s instructions of the FD Rapid Golgi Stain^TM^ Kit (FD Neurotechnologies, Inc., United States) and as we did before (Luo et al., 2020). Briefly, rat brains were incubated in Golgi impregnation solutions for 14 days at 26°C in the dark. The volume of the impregnation solution used for incubation was five times of the brain tissue volume. After impregnation, the brain tissues were transferred into solution C and incubated at 4°C for 7 days in the dark. Coronal brain sections at a thickness of 150 μm and around −2.7 mm from bregma were cut on a vibratome (Microslicer^®^ 10110, Ted Pella, Inc. California, United States). The sections were stained in solution D and solution E. Two to three well individualized neurons in the CA1 region of hippocampus were randomly selected from each rat and sequential optical image stacks of 1388 × 1040 pixels were taken at 1.0 μm intervals along the z-axis (ZEISS, Axio Imager Z2, Germany) with 20× and 60× oil objective. The MBF software (MBF Bioscience, Williston, United States) was used for dimensional reconstruction. The total branch number and dendritic length were measured by Fiji software (Fiji-win64, NIH, United States). The complexity of dendritic trees was estimated using Sholl analysis (Sholl, 1953). For spine density measurement, 2 to 3 neurons were randomly selected from each animal and the matching regions of distal branch dendrites were photographed using a 63× objective (Zhao et al., 2014). The spine numbers in 40 μm segments were counted by an observer who was blind to group assignment. The results were expressed as the number of spines per micrometer segments. The results from one animal were then averaged to reflect the value of each animal. The averaged value of each animal from one group was pooled together for statistical analysis.

### Statistical Analysis

Parametric results in normal distribution are presented as mean ± SD (*n* ≥ 6). The data from the training sessions of Barnes maze test within the same group were tested by one-way repeated measures analysis of variance followed by Tukey test. The data from the training sessions of Barnes maze test between groups were tested by two-way repeated measures analysis of variance followed by Tukey test. All other data were analyzed by one-way analysis of variance followed by the Student-Newman-Keuls test if the data were normally distributed or by one-way analysis of variance on ranks followed by the Student-Newman-Keuls test if the data were not normally distributed. These non-normally distributed data were presented as box plots in the figures. Differences were considered significant at *P* < 0.05 based on two-tailed hypothesis testing. All statistical analyses were performed with SigmaPlot14.0 (Systat Software, Point Richmond, CA, United States).

## Results

No animal died during the surgery or the intended observation period after the surgery. Data from all animals were included for analysis and reported here.

### Amantadine Attenuated Surgery-Induced Learning and Memory Dysfunction in Old Mice

**FIGURE 1:**
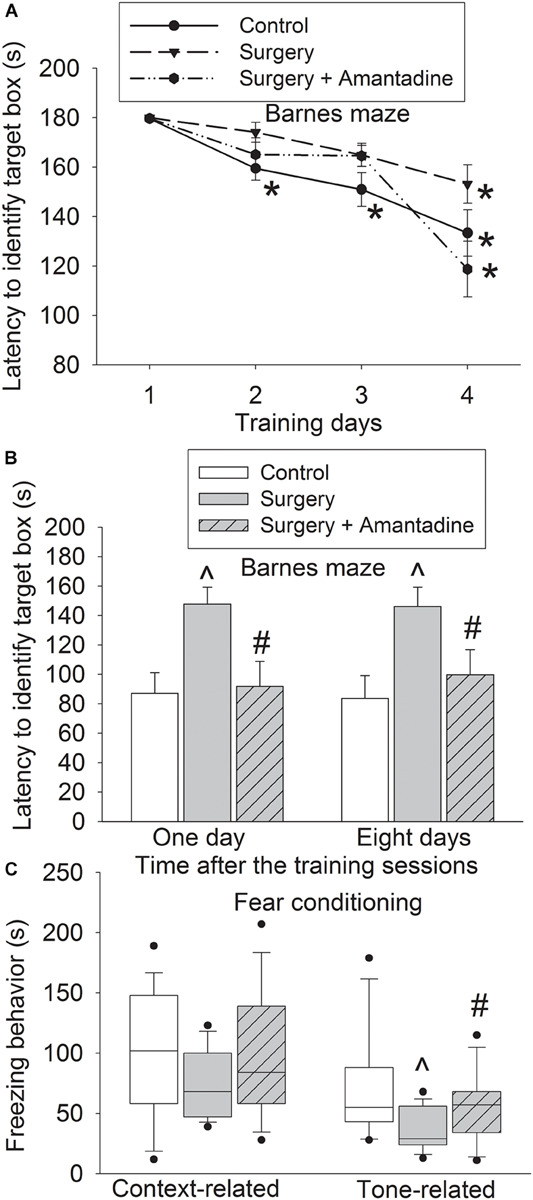
Amantadine attenuated surgery-induced learning and memory impairment in old mice. Old mice were subjected to right carotid exploration under isoflurane anesthesia with or without the treatment of amantadine. Barnes maze training sessions started 1 week after the surgery. **(A)** Barnes maze training sessions. **(B)** Barnes maze memory phase. **(C)** Fear conditioning. Results in panels **(A,B)** are mean ± SEM (*n* = 15). Results in panel **(C)** are in box plot format (*n* = 15). •: lowest or highest score (the score will not show up if it falls in the 95th percentile); between lines: 95th percentile of the data; inside boxes: 25th to 75th percentile including the median of the data. *P < 0.05 compared with the corresponding data of the same animals on day 1. ^∧^*P* < 0.05 compared with control group, ^#^*P* < 0.05 compared with surgery alone group.

The time for old mice to identify the target box was decreased with training no matter whether the mice had surgery or surgery plus amantadine (Figure 1A). Mice needed less time to identify the target box on day 2 to day 4 during the training sessions than they did on day 1 in control group. Mice needed less time to identify the target box on day 4 during the training sessions than they did on day 1 in surgery and surgery plus amantadine group. Surgery was a significant factor to affect the time for mice to identify the target box in the training sessions [*F*(1,28) = 5.039, *P* = 0.033, compared with control group]. This effect was attenuated by amantadine [*F*(1,28) = 0.029, *P* = 0.866, for the comparison between surgery plus amantadine group and control group]. Mice with surgery took longer to identify the target box one day or eight days after the training sessions compared with control group. This effect was attenuated by amantadine (Figure 1B). Surgery reduced freezing behavior in tone-related fear conditioning and this effect was attenuated by amantadine (Figure 1C). These results suggest that surgery induces learning and memory dysfunction, which is attenuated by amantadine.

### GDNF and BDNF Might Play a Role in Amantadine-Reduced Learning and Memory Dysfunction After Surgery in Old Rats

**FIGURE 2:**
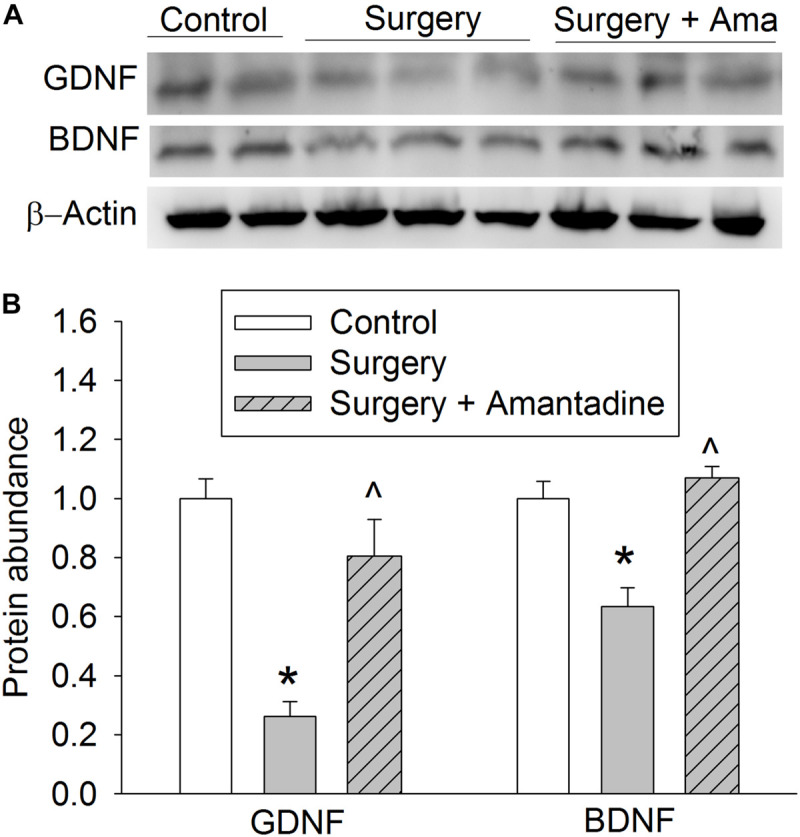
The effects of surgery and amantadine on BDNF and GDNF expression in old rats. Old rats were subjected to right carotid exploration under isoflurane anesthesia with or without the treatment of amantadine. **(A)** Representative Western blotting images. **(B)** Graphic presentation of BDNF and GDNF protein abundance. Results are mean ± SEM (*n* = 7–8). *P < 0.05 compared with control group, ^∧^*P* < 0.05 compared with surgery alone group. Ama: amantadine.

We have shown that surgery reduces GDNF and BDNF expression in young adult rats and mice (Rocha et al., 2012; Zhang et al., 2014). Consistent with the previous findings, surgery reduced GDNF and BDNF expression in the hippocampus of old rats. These effects were attenuated by amantadine (Figure 2).

**FIGURE 3:**
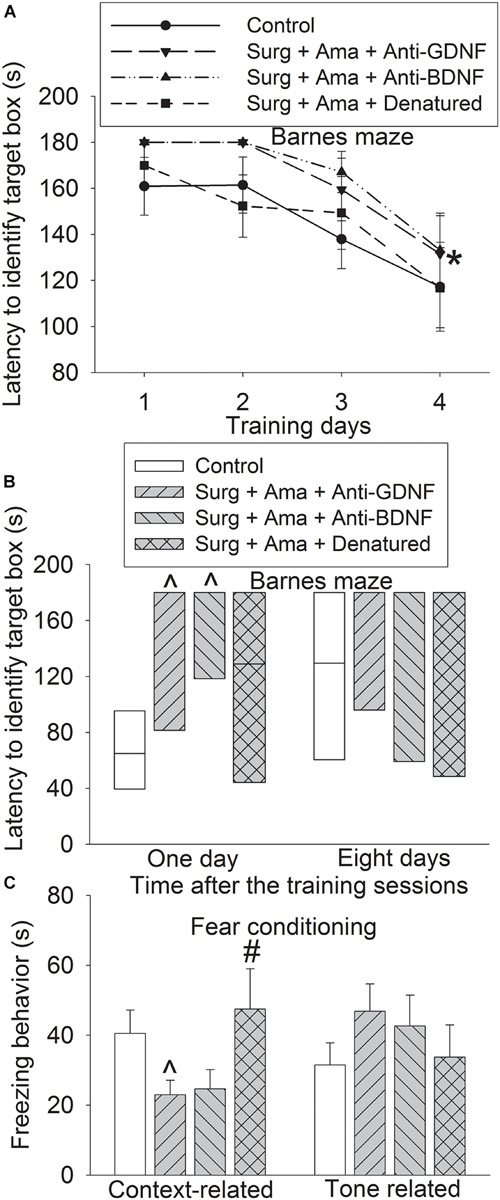
Effects of BDNF and GDNF on learning and memory after surgery in old rats. Rats were subjected to right carotid exploration under isoflurane anesthesia with or without the treatment of amantadine in the presence or absence of intracerebroventricular injection of rabbit polyclonal anti-GDNF antibody, anti-BDNF antibody or heat-denatured anti-GDNF antibody. Barnes maze training sessions started 1 week after the surgery. **(A)** Barnes maze training sessions. **(B)** Barnes maze memory phase. **(C)** Fear conditioning. Results in panels **(A,C)** are mean ± SEM (*n* = 8). Results in panel C are in box plot format (*n* = 8). •: lowest or highest score (the score will not show up if it falls in the 95th percentile); between lines: 95th percentile of the data; inside boxes: 25th to 75th percentile including the median of the data. *P < 0.05 compared with the corresponding data of the same animals on day 1, ^∧^*P* < 0.05 compared with control group, ^#^*P* < 0.05 compared with surgery alone group. Ama, amantadine; surg, surgery.

To determine whether GDNF and BDNF play a role in amantadine-induced protection, old rats received intracerebroventricular injection of GDNF and BDNF. Surgery plus amantadine plus anti-BDNF antibody significantly affected the time needed for rats to identify the target box during the training sessions of Barnes maze test [*F*(1,14) = 4.781, *P* = 0.046, compared with control group]. The effect of surgery plus amantadine plus anti-GDNF antibody was not significant yet during training sessions [*F*(1,14) = 3.193, *P* = 0.096, compared with control group] (Figure 3A). Rats receiving surgery plus amantadine plus anti-GDNF antibody or surgery plus amantadine plus anti-BDNF antibody took longer than control mice to identify the target box in the Barnes maze test on day 1 after the surgery and this effect was attenuated in rats receiving surgery plus amantadine plus heat denatured anti-GDNF antibody (Figure 3B). Rats receiving surgery plus amantadine plus anti-GDNF antibody had reduced context-related freezing behavior and this effect disappeared in rats receiving surgery plus amantadine plus heat denatured anti-GDNF antibody (Figure 3C). These results suggest that anti-BDNF antibody and anti-GDNF antibody blocked the protective effects of amantadine on learning and memory after surgery in old rats.

### GDNF and BDNF Might Play a Role in Amantadine-Reduced Dendritic Arborization Impairment After Surgery in Old Rats

**FIGURE 4:**
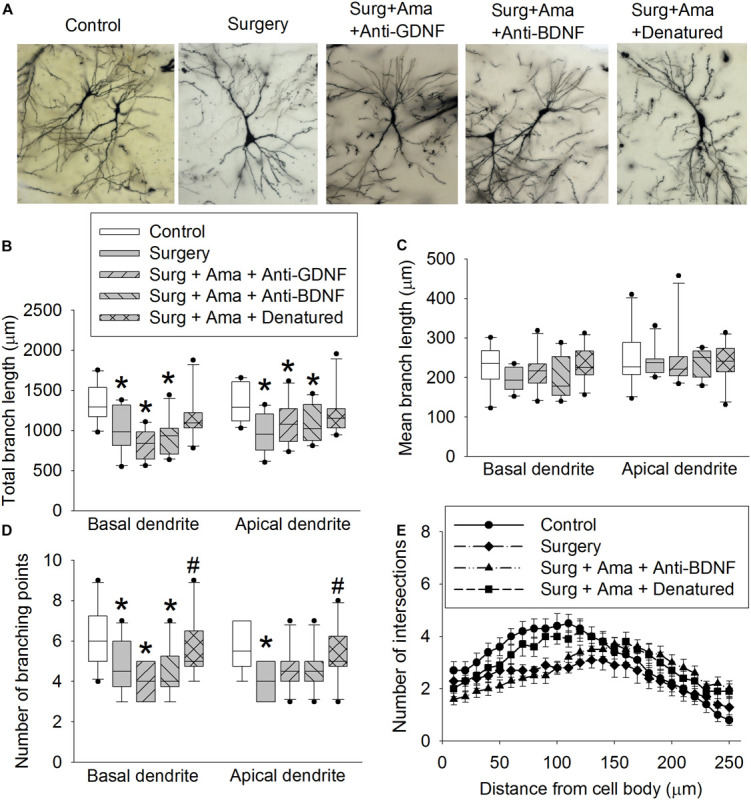
Effects of BDNF and GDNF on dendritic arborization of CA1 pyramidal neurons after surgery in old rats. **(A)** Representative images of neurons. **(B)** Total lengths of basal and apical branches. **(C)** Mean length of basal and apical branches. **(D)** Branch points. **(E)** intersections among branches. Results in panels **(B)** to **(E)** are in box plot format (*n* = 10). •: lowest or highest score (the score will not show up if it falls in the 95th percentile); between lines: 95th percentile of the data; inside boxes: 25th to 75th percentile including the median of the data. *P < 0.05 compared with control group. ^#^*P* < 0.05 compared with surgery alone group. Ama, amantadine; surg, surgery.

**FIGURE 5:**
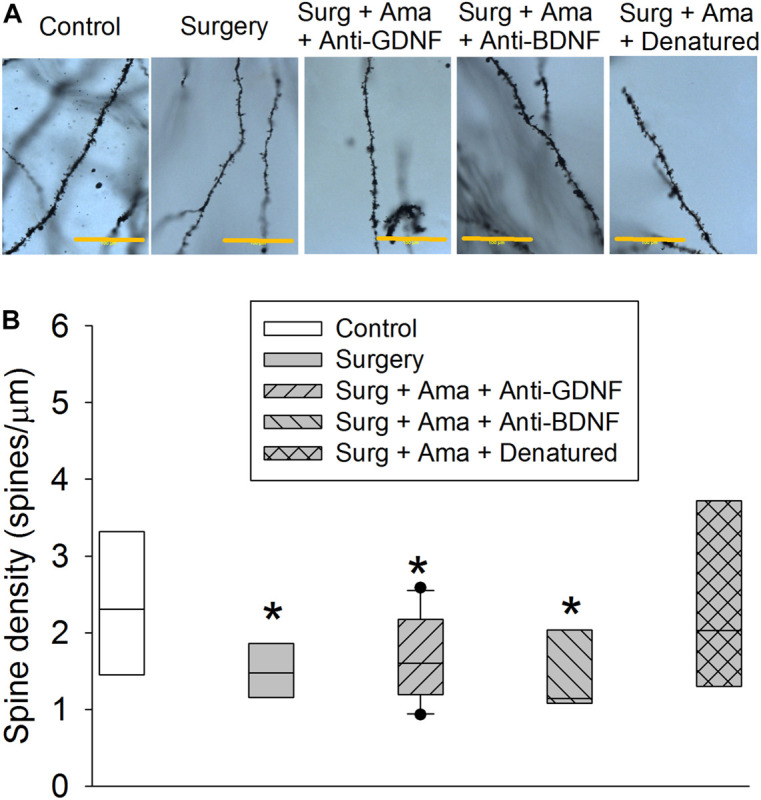
Effects of BDNF and GDNF on spine density of CA1 pyramidal neurons after surgery in old rats. **(A)** Representative images of spines of rats from control, surgery, surgery plus amantadine plus anti-GDNF antibody, surgery plus amantadine plus anti-BDNF antibody or surgery plus amantadine plus heat-denatured anti-GDNF antibody groups, respectively. Scale bar = 100 μm. **(B)** Quantification of spine density. Results in panel **(F)** are in box plot format (*n* = 6–10). •: lowest or highest score (the score will not show up if it falls in the 95th percentile); between lines: 95th percentile of the data; inside boxes: 25th to 75th percentile including the median of the data. *P < 0.05 compared with control group. Ama, amantadine; surg, surgery.

Surgery reduced the total length of basal and apical branches and the number of branches in the hippocampus of old rats. The effect on total length of branches also existed in rats receiving surgery plus amantadine plus anti-GDNF antibody and surgery plus amantadine plus anti-BDNF antibody but was not present in rats receiving surgery plus amantadine plus heat denatured anti-GDNF antibody. The reduction on the number of basal and apical branches by surgery was attenuated in rats receiving surgery plus amantadine plus heat-denatured anti-GDNF antibody. None of the conditions affected the mean length of basal branch (Figures 4A–D). Surgery was a significant factor to affect the number of intersections among branches [*F*(1,18) = 5.238, *P* = 0.034, compared with control group]. This effect was not present in rats receiving surgery plus amantadine plus heat denatured anti-GDNF antibody [*F*(1,18) = 0.001, *P* = 0.979, compared with control group] (Figure 4E). Similar to the results of dendritic arborization, spine density in the hippocampus of old rats was decreased in rats with surgery or receiving surgery plus amantadine plus anti-GDNF antibody or surgery plus amantadine plus anti-BDNF antibody but not in rats receiving surgery plus amantadine plus heat denatured anti-GDNF antibody (Figure 5). These results suggest that surgery impairs dendritic arborization and that this impairment was attenuated by amantadine.

## Discussion

We have shown that amantadine attenuates POCD in young adult rats (Zheng et al., 2017) and sepsis-induced encephalopathy in young adult mice (Xing et al., 2018). However, age is a risk factor for POCD (Newman et al., 2001; Monk et al., 2008). The current study showed that amantadine attenuated learning and memory dysfunction in old mice and rats, providing initial evidence of the protection against POCD in age-relevant animals.

Consistent with our previous studies in young animals (Fan et al., 2016; Gui et al., 2017; Zheng et al., 2017), surgery reduced GDNF and BDNF expression in old rats. This reduction was attenuated by amantadine. Since our previous studies have shown that GDNF and BDNF may play a role in POCD in young adult rodents (Fan et al., 2016; Gui et al., 2017; Zheng et al., 2017), the effects of maintaining GDNF and BDNF levels in the brain may be a mechanism for amantadine to reduce POCD. Our results showed that anti-GDNF antibody and anti-BDNF antibody blocked amantadine effects on learning and memory in old rats with surgery. Thus, the reduced BDNF and GDNF in rats may be important for the development of POCD and preserving the expression of BDNF and GDNF may be a mechanism for amantadine-induced protection against POCD.

BDNF and GDNF are known to play a role in maintaining neuronal structural integrity (Guan et al., 2009; Irala et al., 2016). Consistent with this idea, surgery decreased BDNF and GDNF expression. Surgery also decreases the number of dendritic branches, total length of dendritic branches, dendritic intersections and spine density. Amantadine attenuated these effects. In addition, anti-BDNF and anti-GDNF antibodies blocked amantadine effects. These results suggest that GDNF and BDNF play an important role in surgery-induced impairment of dendritic arborization and that preserving the expression of GDNF and BDNF is important in the effects of amantadine on dendritic arborization in old rats after surgery. Since dendritic arborization and spine density are structural basis for learning and memory (Gillani et al., 2010; Knutson et al., 2016), maintaining the integrity of dendritic arborization and spine density shall be a process downstream of preserving BDNF and GDNF expression for amantadine-induced protection against POCD.

One important question is how surgery impairs the expression of BDNF and GDNF. It is known that surgery induces neuroinflammation (Cao et al., 2012; Zhang et al., 2014; Zheng et al., 2017). Our study has shown that inflammation impairs the expression of GDNF after surgery (Gui et al., 2017) and surgery also activates histone deacetylases, a group of enzymes involved in epigenetic regulation of gene expression (Guan et al., 2009), to decrease BDNF expression (Luo et al., 2020). Inflammation can activate histone deacetylases (Correa et al., 2011). Thus, it is possible that surgery induces neuroinflammation that activates epigenetic regulation mechanisms, such as histone deacetylases, to decrease the expression of BDNF and GDNF. Since amantadine can reduce neuroinflammation (Zhang et al., 2014), it is possible that amantadine inhibits inflammation and, therefore, blocks the activation of epigenetic regulation of gene expression induced by surgery. Additional experiments are needed to determine whether this pathway is the mechanism for amantadine to maintain BDNF and GDNF expression in animals with surgery.

We performed learning and memory tests between 1 and 3 weeks after surgery to determine these functions of rodents at a delayed phase. Animals during this time period shall not have significant pain at surgery site that can affect their performance during learning and memory tests. Brain tissues were harvested 3 days or 3 weeks after surgery for Western blotting analysis or Golgi staining, respectively, because our previous studies have shown growth factors have a changed expression by 3 days after surgery in young rodents (Zhang et al., 2014; Gui et al., 2017). A delayed time when animals remained to have learning and memory dysfunction was chosen to examine brain structural changes (Golgi staining examination) to identify structural bases for the dysfunction of learning and memory.

Our findings may have clinical implication. Amantadine has been used clinically. We have now shown the effectiveness of amantadine in attenuating POCD in age-relevant models of two animal species. If the effectiveness of amantadine in protecting patients against POCD is confirmed, using amantadine in the perioperative period may be a practical approach for reducing POCD.

Our study has limitations. Old animals are precious resource that is very limited in amount. Since we have shown that surgery induces learning and memory dysfunction in old rodents (Bi et al., 2017) and in old mice in this current study, we did not include surgery alone group in the learning and memory study of old rats. In addition, heat shall inactivate the anti-GDNF antibody as we showed before (Zhang et al., 2014; Gui et al., 2017). We included surgery plus amantadine plus heat-inactivated anti-GDNF antibody group and did not have surgery plus amantadine group in the learning, memory, dendritic arborization, and spine density studies because the results of these two groups shall be very similar. These experimental designs are in accordance with the principle of using the minimal number of animals to maximize the outcome. Another limitation is that we showed that surgery reduced BDNF and GDNF expression. It is not known whether other growth factors are affected. Also, only male mice and rats were used in this study due to limited access to old rodents. Old female rodents shall be used in the future studies to test the effects of amantadine. Finally, our previous studies did not show that amantadine had an effect on learning and memory of control rats or mice (Zhang et al., 2014; Xing et al., 2018). Amantadine alone group was not included in rat or mouse studies to reduce the number of animals needed for the studies. Although a study with 4 groups (control, amantadine alone, surgery, and surgery plus amantadine) can determine the interaction of amantadine and surgery by using two-way analysis of variance, this determination may not be critical because one of our study goals was to investigate whether amantadine improved the learning and memory of old rodents with surgery.

In summary, our results suggest that amantadine attenuates POCD. This effect may be mediated by preserving BDNF and GDNF expression, which maintains dendritic arborization and spine density to protect the learning and memory of old rodents.

## Data Availability Statement

The raw data supporting the conclusions of this article will be made available by the authors, without undue reservation.

## Ethics Statement

The animal study was reviewed and approved by the institutional Animal Care and use Committee of the University of Virginia (Charlottesville, VA, United States).

## Author Contributions

ZZ conceived the project and performed the final data analysis and wrote the manuscript. JZ and ZZ designed the study. JZ and JL performed the experiments. JZ and CN did the initial the data analysis. JZ drafted “Methods and Materials” section. All authors contributed to the article and approved the submitted version.

## Conflict of Interest

The authors declare that the research was conducted in the absence of any commercial or financial relationships that could be construed as a potential conflict of interest.
